# Mucinous adenocarcinoma of the intestinal type arising from mature cystic teratoma of the ovary: a rare case report and review of the literature

**DOI:** 10.1186/1757-2215-5-41

**Published:** 2012-12-05

**Authors:** Masaaki Takai, Masanori Kanemura, Hiroshi Kawaguchi, Satoe Fujiwara, Saha Yoo, Yoshimichi Tanaka, Satoshi Tsunetoh, Yoshito Terai, Takashi Yamada, Masahide Ohmichi

**Affiliations:** 1 Department of Obstetrics and Gynecology, Osaka Medical College, 2-7, Daigaku-machi, Takatsuki, Osaka, 569-8686, Japan; 2 Department of Pathology, Osaka Medical College, Osaka, Japan

**Keywords:** Malignant transformation, Mature cystic teratoma, Adenocarcinoma, Intestinal type

## Abstract

**Background:**

Mature cystic teratomas (MCTs) are the most common germ cell tumors of the ovary. Malignant tranformation occurs in 1-2% of these neoplasms. Although most of the malignancies arising from MCTs are squamous cell carcinomas, adenocarcinoma of the gastrointestinal type is extremery rare. We herein present a case of adenocarcinoma of the intestinal type arising from a MCT.

**Case:**

A 49-year-old female underwent surgery for a left ovarian tumor. The histology of the cyst walls revealed a MCT with a few hair shafts and a squamous layer, while another part of the tumor showed adenocarcinoma of the intestinal type. Five years after surgery, she is alive without disease.

## Background

Mature cystic teratomas (MCTs) are the most common germ cell tumors of the ovary. MCTs comprise 18% of ovarian neoplasms, and malignant teranformation occurs in 1-2% of these neoplasms [1] . Most of the malignancies arising from MCTs are squamous call carcinomas, with adenocarcinomas comprising only 7% of the malignant tumors [2]. Moreover, there have been few reports of adenocarcinoma of the gastrointestinal type in the ovary arising from a MCT [2-5]. We herein present a case of ovarian adenocarcinoma of the intestinal type arising from a MCT in a perimenopausal female, and provide a review of the literature.

### Case presentation

**Figure 1:**
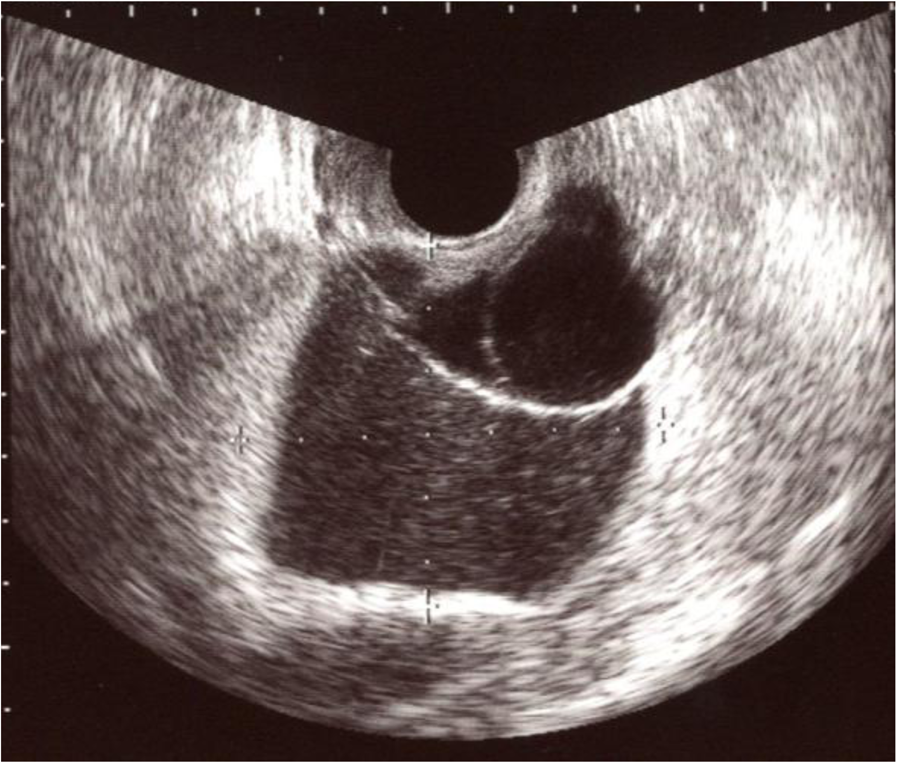
**Transvaginal ultrasonography.** Transvaginal ultrasonography showed a multilocular cystic lesion without a solid part in the pelvic cavity.

**Figure 2:**
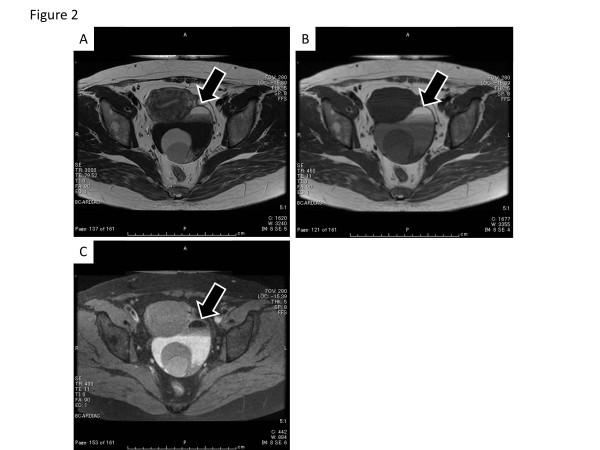
**MRI findings before surgery.** Horizontal T2-weighted (**A**) and T1-weighted (**B**) MR imaging demonstrated a fat component, which showed a drop in the signal intensity on fat saturated T1-weighted images (**C**) in the multilocular cystic lesion without a solid part (arrow).

A 49-year-old female, gravida 5, para 3, presented with dysuria and hematuria. Her past medical, surgical, gynecological, and family histories were all unremarkable. Her menstrual cycle was irregular. A bimanual pelvic examination identified a movable mass in the left adnexal region. Vaginal ultrasound confirmed the presence of a polycystic mass of 67 x 57 mm in diameter without a solid component, suggestive of ovarian tumor (Figure 1). Ascites was absent, and there were no abnormal findings of the uterus and right adnexae. Magnetic resonance imaging (MRI) showed a multilocular cystic mass with a fat component (Figure 2). The serum tumor marker levels were within the normal ranges: CA125; 20.0U/mL (normal <35.0), CA19-9; 3.8 U/mL (normal <37.0), SCC; 1.1ng/mL (normal <1.5), except for carcinoembrionic antigen (CEA); 6.9ng/mL (normal <5.0). The diagnosis of left ovarian MCT was made based on these data, and laparoscopic surgery was planned.

An exploratory laparoscopy revealed an enlarged left ovary (80mm × 70mm). The surface of the tumor was smooth and well-circumscribed. There was no adherence to any other pelvic organs. A small amount of ascites was detected, and the cytology of ascites was negative for malignancy. A laparoscopic left salpingo-oophorectomy was performed, and the intraoperative diagnosis of frozen sections was suspicious of borderline malignancy. Therefore, we changed the procedure to a laparotomy and abdominal simple total hysterectomy, and a right salpingo-oophorectomy and partial omentectomy were carried out. The patient’s postoperative course was uneventful. The pathological findings revealed the tumor to be adenocarcinoma of the intestinal type arising from a MCT. The serum level of CEA was normalized 7 days after surgery. We explained to the patient that there was no evidence about the need for adjuvant therapy because the disease is extremely rare. She decided not to receive adjuvant therapy. The patient had no recurrence of the disease as of 5 years after the surgery.

### Macroscopic and microscopic evaluations

**Figure 3:**
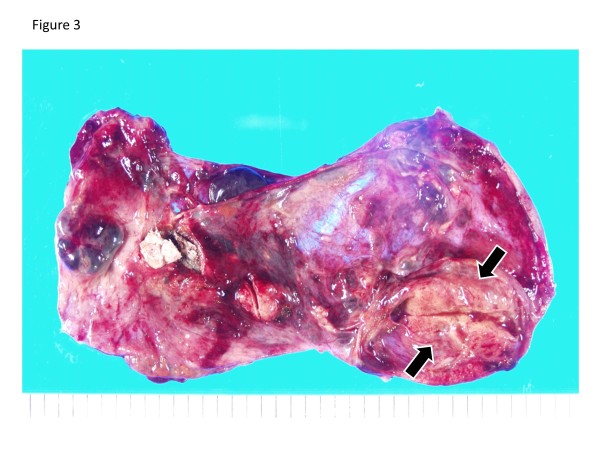
**The gross appearance of the tumor.** A 3 cm slightly yellowish solid part (arrows) was observed.

**Figure 4:**
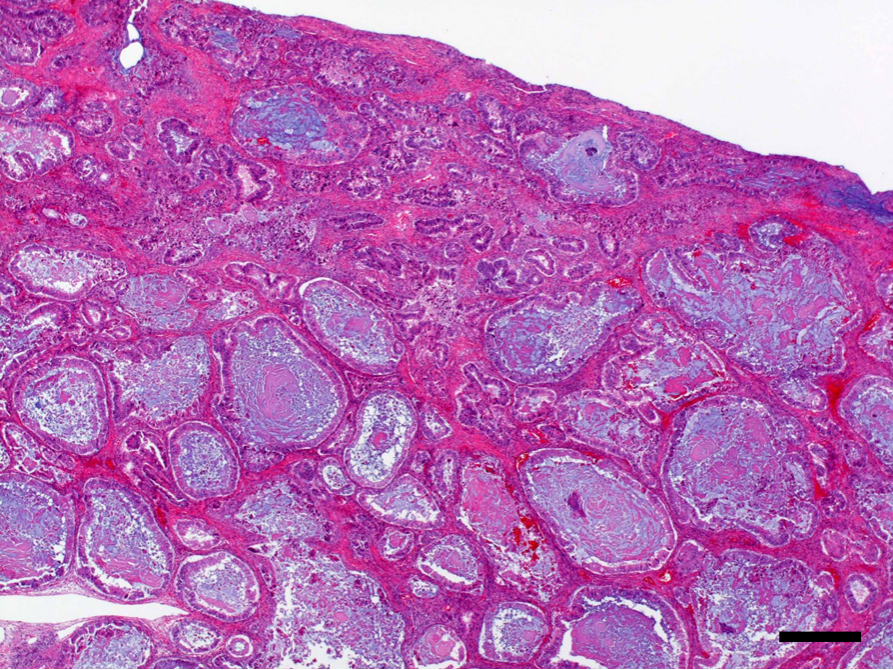
**Microscopic findings**[1]**.** The cells which formed the lumen of cysts were mostly columnar epithelium, with some goblet cells. (H&E x20, bar = 500 μm).

**Figure 5:**
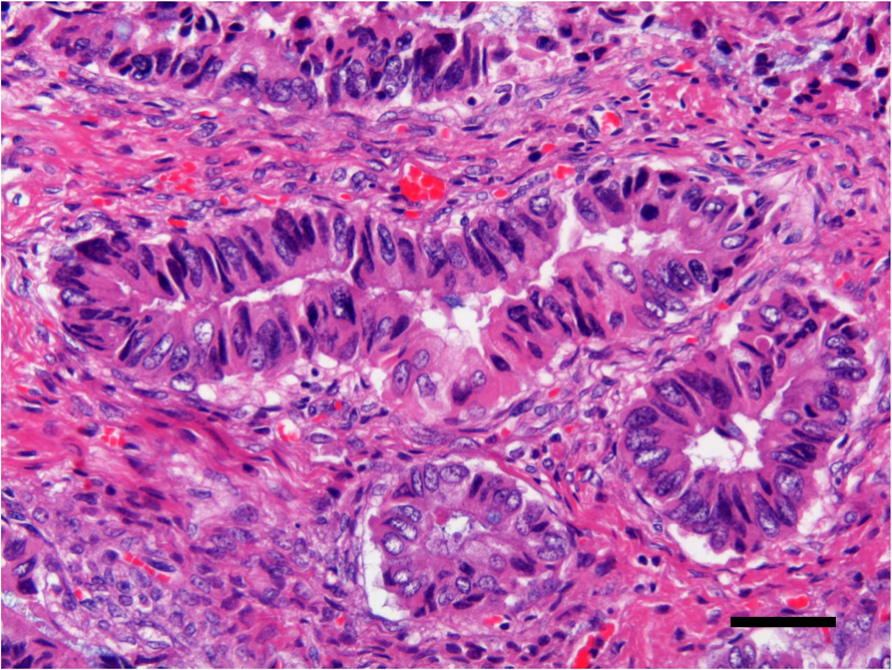
**Microscopic findings**[2]**.** The consistency of the small glandular cavity was high, and severe dyskaryotic cells had become multilayered, with stromal invasion. (H&E x200, bar = 50 μm).

Macroscopically, the resected ovarian mass was cystic and contained sebaceous material, hair, and calcification. The inner surface of the mass was rough, and had partial thicking-like nodules (Figure 3). The gross appearance of the uterus, right adnexae and omentum were normal. The microscopic examination of the ovarian mass revealed that the formation contained a lot of large and small cysts including mucin, cell debris and calcification. The cells which formed the lumen of cysts were mostly columnar epithelium, with some goblet cells (Figure 4). The consistency of the small glandular cavity was high, and severe dyskaryotic cells had become multilayered. The nuclei were enlarged and irregularly shaped and contained coarse chromatin, multidistributed and showed atypia. Most nuclei had a large and brightly nucleoli. In some areas, the malignant transformation of the intestinal epithelium resulted in a well-differentiated adenocarcinoma with stromal invasion (Figure 5).

**Figure 6:**
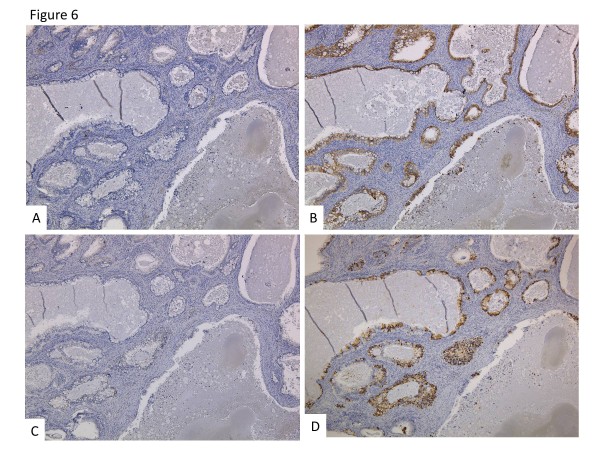
**Immunohistchemical staining.** CK7 (**A**) and Muc-5AC (**C**) were negative. CK20 (**B**) and Muc-2 (**D**) were positive.

**Figure 7:**
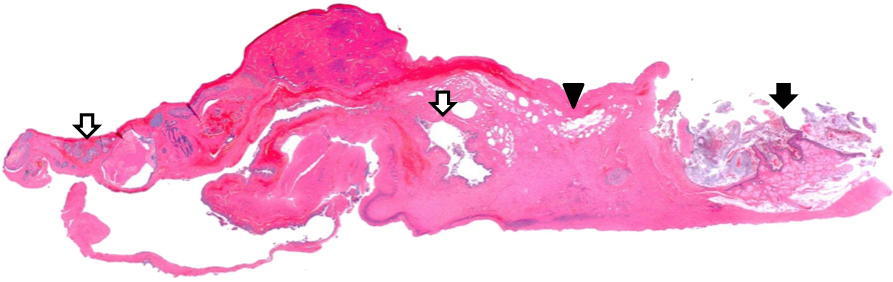
**Microscopic cross-section through the tumor.** The tumor contained components of squamous layer, sebaceous gland (black arrow), fat tissue (arrow head) and atypical grand (white arrow). (H&E x4).

In the immunohistochemical staining studies, CK20 (Dako) and Muc-2 (Novocastra) were positive, and CK7 (Dako), Muc-5AC (Novocastra) (Figure 6), Muc-6 (Novocastra), the ER (Dako), and the PR (Dako) (Figure not shown) were negative in the adenocarcinomatous part. Another part of the cyst wall revealed a MCT with a few hair shafts, squamous layer and sebaceous gland (Figure 7). There were no immature components in the tumor.

The final diagnosis was left ovarian MCT with malignant transformation and mucinous adenocarcinoma, intestinal type, FIGO stage Ic (b).

## Conclusions

Malignant transformation of MCTs has been recorded as occurring in 1-2% of cases [1]. The most common form of malignant transformation of MCTs is squamous cell carcinoma, which occurs in about 75% of cases, while adenocarcinoma arising from MCTs is rare, with an incidence of 7% [6]. Adenocarcinoma of the gastrointestinal type is extremely rare. The immunohistochemical analysis showed positive staining for CK20 and Muc-2, while CK7, Muc-5AC and Muc-6 were negative in the adenocarcinomatous part. Therefore, we diagnosed the tumor as adenocarcinoma of the intestinal type arising from a MCT. To our knowledge, only six cases of adenocarcinoma of the gastrointestinal type arising from MCTs have been reported in the English literature [2-7]. The relevant clinical and pathological findings of these cases are summarized in Table 1. The patients’ ages ranged from 37 to 77 years old (mean, 50 years). All of the cases were of stage I disease, excluding the second patient. All patients had undergone surgery and had been diagnosed based on the postoperative histological findings. Two patients received adjuvant chemotherapy, but one patient died of disease 3 months after surgery.

**Table 1:** Preveous reports of adenocarcinoma of the gastrointestinal type in the ovary arising from a MCT

**Case**	**Source**	**Age**	**Tumor marker**	**Tumor size (cm)**	**Surgical procedure**	**FIGO stage**	**Adjuvant therapy**	**Follow up**
1	G.Ueda et al. (1993)	62	N.E.	35	TAH+BSO	Ia	FAMT	15 years
2	Fishman A et al. (1998)	38	CEA: 40 ng/ml CA 125: 80 U/ml CA153: 60 U/ml	20 x 13 x 8.5	TAH+BSO+OMT +appendectomy	IIIc	5FU Leucovorin	DOD 3 month after surgery
3	Levine DA et.al (2004)	37	CEA: 11.2 ng/ml CA 125,AFP, HCG: WNL	15 x 12 x 11	Unilateral SO +OMT +PLN+PAN	Ia	none	40 month
4	Kushima M (2004)	52	CA19-9: 109 U/ml SLX: 58.5 U/ml CA125: 36 U/ml CA72-4: 19 U/ml	6.4 x 4.8 x 2.8	Bilateral SO	Ia	none	31 month
5	KJ Min et.al (2006)	77	CA125: 72 U/ml	17 x 14 x 2	TAH+BSO	Ia	none	12 month
6	Gunney M et.al (2006)	38	CEA: WNL CA1 25: 99.1 U/ml CA19-9: >1000 U/ml	N.D.	TAH+BSO+OMT +PLN+PAN	Ia	none	N.D.
7	present case	49	CEA: 6.9 ng/ml CA 125: 20 U/ml CA19-9: 3.8 U/ml SCC: 1.1 ng/ml	6.7 x 5.7	TAH+BSO+OMT	Ic(b)	none	5 years

*1 TAH: total abdominal hysterectomy, SO: salpingo-oophorectomy, OMT: omentectomy, PLN: pelvic lymphadenecomy, PAN: para aortic lymphadenectomy, *2 FAMT:5-furuorouracil+endoxan+mitomycin-C+toyomycin.

*3 N.E.: not examined *4 N.D.: no discription WNL : within normal limits.

MCTs of the ovary represent the majority of benign ovarian neoplasms in females younger than 30 years of age [8], whereas malignant transformation of MCTs is usually found in postmenopausal females. The present case was perimenopausal and three of the six previous cases were premenopausal. Adenocarcinoma of the gastrointestinal type arising from MCTs may occur at a lower age than other histological types of malignant transformation of MCTs.

Recently, laparoscopic surgery for benign ovarian cystic tumors has been performed rather than laparotomy. However, there have been concerns about artificial tumor rupture due to chemical peritonitis and the spread of tumor cells. Hackethal et al. [9] reviewed and analyzed the published data about squamous cell carcinoma arising from MCTs of the ovary. According to their data, unlike common ovarian carcinomas, rupture of the tumor capsule has no adverse prognostic effects. In the present case, we performed a laparoscopic salpingo-oophorectomy and could not avoid the artificial rupture of the tumor. Fortunately, our case had no recurrence in the abdominal cavity. However artificial rupture of the tumor should be avoided in order to prevent recurrence [9]. Furthermore, the previous analysis revealed that while hysterectomy, bilateral salpingo-oophorectomy and lymphadenectomy were associated with a better outcome, omentectomy did not affect the overall survival. This may suggest that the spreading pattern of malignant transformation of MCTs differs from that of epithelial ovarian cancer.

Several reports suggested that the SCC antigen, either alone or in combination with other markers, such as macrophage-colony stimulating factor (M-CSF) and carcinoembryonic antigen (CEA), should be considered useful markers for squamous cell carcinoma arising from MCTs for making a preoperative diagnosis [10-12]. On the other hand, there has been no data regarding adenocarcinoma arising from MCTs because of the small number of cases. However, as shown in Table 1, an elevation of CEA was seen in three of the 7 cases, including our present case. Therefore, tumors markers may be useful for making a preoperative diagnosis.

MCTs might occur as a result of failure of the first meiotic division, but it is unclear how the malignant transformation of MCTs occurs. Rim et al. [13] studied 11 cases of malignant transformation of MCTs and elucidated that 80% of MCTs are diagnosed in subjects who are of reproductive age. Based on that finding, Amanjit et al. [14] suggested that the malignant transformation of MCTs could be related to the long-term presence of non-removed MCTs. However, the pathogenesis is still unknown, and it is unclear whether malignant transformation occurs separately or co-exists with carcinoma.

It is difficult to make a diagnosis of malignant transformation of a MCT preoperatively. MRI findings may be helpful to distinguish malignant transformation from benign neoplasms. Several reports have revealed that an important feature of the malignant transformation of MCTs was the existence of an enhanced solid component in MCTs [15-18]. In our case, MRI was performed without gadolinium enhancement, therefore, it was difficult to determine whether the lesion was malignant.

When malignant transformation of MCTs is suspected preoperatively, enhanced MRI, intraoperative consultation and a postoperative histological diagnosis are very important.

### Consent

Written informed consent was obtained from the patient for publication of this Case Report and any accompanying images. A copy of the written consent is available for review by the Editor-in-Chief of this journal.

## Competing interests

The authors declare that they have no competing interests.

## Authors’ contributions

MT, MK, YT and MO carried out surgery of the patient and drafted the manuscript. TY, MK, YT, ST and MT examined microscopically surgical specimen and performed the immunohistochemical staining of the tumor. MT, MK, FS and SY reviewed papers about the MCTs. MT, MK and MO conceived of the study, and participated in its design and coordination and helped to draft the manuscript. All authors read and approved the final manuscript.
